# PI3K-AKT Signaling via Nrf2 Protects against Hyperoxia-Induced Acute Lung Injury, but Promotes Inflammation Post-Injury Independent of Nrf2 in Mice

**DOI:** 10.1371/journal.pone.0129676

**Published:** 2015-06-15

**Authors:** Narsa M. Reddy, Haranatha R. Potteti, Suryanarayana Vegiraju, Hsin-Jou Chen, Chandra Mohan Tamatam, Sekhar P. Reddy

**Affiliations:** 1 Department of Pediatrics, University of Illinois at Chicago, Chicago, Illinois, United States of America; 2 Department of Environmental Health Sciences, Johns Hopkins Bloomberg School of Public Health, Baltimore, Maryland, United States of America; University of Pittsburgh, UNITED STATES

## Abstract

Lung epithelial and endothelial cell death accompanied by inflammation contributes to hyperoxia-induced acute lung injury (ALI). Impaired resolution of ALI can promote and/or perpetuate lung pathogenesis, including fibrosis. Previously, we have shown that the transcription factor Nrf2 induces cytoprotective gene expression and confers protection against hyperoxic lung injury, and that Nrf2-mediated signaling is also crucial for the restoration of lung homeostasis post-injury. Although we have reported that PI3K/AKT signaling is required for Nrf2 activation in lung epithelial cells, significance of the PI3K/AKT-Nrf2 crosstalk during hyperoxic lung injury and repair remains unclear. Thus, we evaluated this aspect using *Nrf2* knockout (*Nrf2* ^–/–^) and wild-type (*Nrf2* ^+/+^) mouse models. Here, we show that pharmacologic inhibition of PI3K/AKT signaling increased lung inflammation and alveolar permeability in *Nrf2* ^+/+^ mice, accompanied by decreased expression of Nrf2-target genes such as Nqo1 and Hmox1. PI3K/AKT inhibition dampened hyperoxia-stimulated Nqo1 and Hmox1 expression in lung epithelial cells and alveolar macrophages. Contrasting with its protective effects, PI3K/AKT inhibition suppressed lung inflammation in *Nrf2* ^+/+^ mice during post-injury. In *Nrf2* ^–/–^ mice exposed to room-air, PI3K/AKT inhibition caused lung injury and inflammation, but it did not exaggerate hyperoxia-induced ALI. During post-injury, PI3K/AKT inhibition did not augment, but rather attenuated, lung inflammation in *Nrf2* ^–/–^ mice. These results suggest that PI3K/AKT-Nrf2 signaling is required to dampen hyperoxia-induced lung injury and inflammation. Paradoxically, the PI3K/AKT pathway promotes lung inflammation, independent of Nrf2, during post-injury.

## Introduction

Lung alveolar epithelial and endothelial cell death accompanied by inflammation is a major hallmark of acute lung injury (ALI) caused by oxidant and pro-fibrotic agents. Impaired resolution of ALI increases susceptibility to subsequent exposure to various insults and/or promotes pathogenesis, including lung fibrosis [1, 2]. Cellular stress due to an imbalance between anti-oxidants and oxidants and anti- and pro-inflammatory cytokines plays a key role in lung disease progression and exacerbation [3–5]. The transcription factor NF-E2-related factor 2 (Nrf2) modulates redox homeostasis and lung inflammation through the regulation of anti-oxidant and cytokine gene expression in response to injurious insults. We have shown that Nrf2-deficiency causes greater susceptibility to hyperoxia-induced ALI [6, 7] and impairs the resolution of inflammation during recovery in mice [8], due to diminished levels of both basal and inducible expression of proteins required for the detoxification of excessive reactive electrophiles generated by hyperoxia [9].

The phosphatidylinositol 3’-kinase (PI3K)/AKT signaling pathway regulates cell survival during oxidative stress [10–13] and confers protection against oxidant-induced lung injury in rodents [13]. Previously, we have shown that the PI3K/AKT pathway regulates Nrf2 activation by hyperoxia in lung epithelial cells [11, 14, 15]. Other studies have shown that the inhibition of PI3K-γ signaling ameliorates LPS- and ventilator-induced lung injury through the regulation of the IκBα/NF-κB pathway [16, 17]. Genetic ablation of PI3K-γ enhanced neutrophil sequestration in experimental sepsis [18], and PI3K/AKT inhibitor LY294002 suppressed inflammatory responses in experimental asthma [19]. These results suggest the PI3K/AKT pathway regulates lung injury and inflammation in a contextual manner.

To examine whether PI3K/AKT signaling modulates hyperoxia-induced ALI through Nrf2 *in vivo*, here we studied the effects of PI3K/AKT inhibition on hyperoxic lung injury and repair using Nrf2-suffcient (wild-type or *Nrf2* ^+/+^) and Nrf2-deficient (*Nrf2* ^–/–^) mouse models. Here, we show that PI3K/AKT inhibition during hyperoxia dampens Nrf2-target gene expression with a concomitant increase in lung injury and inflammation, suggesting that PI3K/AKT imparts its protective functions via Nrf2. We also report that the PI3K/AKT pathway promotes lung inflammation during the post-injury period, independent of Nrf2.

## Methods

### Mice and hyperoxia exposure

The wild-type (*Nrf2*-sufficient or *Nrf2* ^+/+^) and *Nrf2*-deficient (*Nrf2* ^–/–^) mice (CD-1/ICR strain, 8-week-old, females, 25–30 g) were administered with LY294002 (5 mg/kg BW) or vehicle intraperitoneally. LY294002 was dissolved in DMSO (15 mg/ml) and 125 μg LY294002 in 8.4 μl was diluted to 200 μl with PBS. Similarly vehicle, 8.4 μl DMSO diluted to 200 μl with PBS, was prepared. Mice were injected with LY294002 or vehicle, and after one hour they were exposed to hyperoxia or room air as previously described [8]. Mice were housed 5 per cage, and cages were placed in a hyperoxia exposure chamber (Cat # A30274, Biospherix Ltd, NY, USA,). The chamber bottom was lined with sufficient CO_2_ absorbent (Soda-sorb; WR Grace, Lexington, MA). Food and water were provided *ad libitum*. Sufficient humidified oxygen (Airgas, Baltimore) was delivered to the chamber continuously and the oxygen concentration was adjusted to 95–98% and monitored with Pro-Ox monitor (Model E702, Biospherix). After 24 h, mice were injected with LY294002 and exposed to hyperoxia for another 24 h. In a second set of experiments, mice exposed to 48 h hyperoxia were immediately treated with vehicle or LY294002 and then allowed to recover in room air for 72 h. During recovery mice were treated with LY294002 or vehicle at 24 h and 48 h. Note that all the animal work in this study was performed at the PI's (SPR) laboratory in the Department of Environmental Health Sciences, the Johns Hopkins Bloomberg School of Public Health.All the animal protocols were approved by the animal care and use committee of the Johns Hopkins University.

### Cell culture and hyperoxia exposure

MH-S (a murine alveolar macrophage cell line) (kindly provided by Dr. Irena Levitan), and MLE-12 (murine lung alveolar type II-like cell line) (kindly provided by Dr. Jeff Whitsett) cells were cultured in RPMI 1640 and DMEM medium, respectively, and supplemented with 10% FBS, 1% antibiotics and fungizone. Cells were treated with vehicle or LY294002 (10 μM) for 30 minutes and then exposed to room air or hyperoxia. Cells were placed in modular chambers (MC101, Billups-Rothenberg Inc., Del Mar, CA) and chambers were equilibrated with a gas mixture of 95% O_2_ + 5% CO_2_ for 10 min. After equilibration, the gas connection was disconnected and chambers were placed in a 37°C incubator for the desired time periods. The control cultures were incubated at 37°C in an atmosphere of 5% CO_2_.

### Transient transfection of AKT1 siRNA

Suppression of AKT1 function was performed by knockdown of AKT1 expression by AKT1 small interfering RNA (AKT1-siRNA) (cat# M-004364-00, Cell Signaling Technology). Transient transfections were done with cells at 60% confluence using Dharmafect siRNA transfection reagent according to the manufacturer’s (Thermo Scientific) instructions. Briefly, MLE-12 cells were seeded in six-well plates at 1.5×10^5^ cells/well and incubated at 37°C for 24 h. Each of AKT1-siRNA or non-target control siRNA (100 nmol) was transfected into the cells with 7.5 μl of Dharmafect reagent in serum-free media without antibiotics and incubated for 6 h at 37°C. After removal of siRNA transfection reagent, medium with fetal bovine serum was added and incubated further for 18 h. Cell cultures were subsequently exposed to room air or hyperoxia. The efficacy of AKT1 knockdown and its effects on Nrf2 target gene expression were assessed by real-time RT-PCR analysis.

### Assessment of lung injury and inflammation

After exposure, lung injury was assessed by alveolar permeability, whereas lung inflammation was evaluated by differential cell counts in bronchoalveolar lavage (BAL) fluid obtained from the right lung [8]. The left lung was inflated to 25 cm of water pressure and fixed with 0.8% low-melting agarose in 1.5% buffered paraformaldehyde for 24 h, and 5 μm lung sections were cut and stained with hematoxylin and eosin (H&E). The BAL fluids were centrifuged and the supernatant was stored at -80°C. BAL fluid protein concentration was measured by Bio-Rad protein assay reagent (Cat # 500–0006, Bio-Rad, Hercules, CA). Differential cell counts were performed after staining the cells with Diff-Quik stain kit (Cat # B4132- 1A, Dade Behring, DE, USA).

### Western blot analysis

Total proteins were extracted from lung tissues and cells in a lysis buffer consisting of 20 mM Tris (pH 7.5), 150 mM NaCl, 1 mM EDTA, 1 mM EGTA, 1% Triton X-100, 2.5 mM sodium pyrophosphate, 1 mM Na_3_VO_4_, 5 mM β-glycerophosphate, and 1 μg/ml leupeptin. Comparable amounts of total protein (~40 μg) from each sample were separated and membranes were probed with antibodies specific for phospo-AKT (Cat # 2965), AKT (Cat # 4691) (Cell Signaling Technology, Baverly, MA), Hmox1 (Santa Cruz Biotech, Santa Cruz, CA), or Nqo1 (Abcam, Cambridge, UK). β-actin (Sigma, St. Louis, MO) was used as reference. The blots were developed using a HyGlo-ECL kit (Denville Scientific Inc, Metuchen, NJ).

### Statistical Analyses

Data were expressed as the mean ± SEM. “*p”* value of ≤ 0.05 is considered significant and was determined using the Student’s *t*-test. For studies, related to lung injury and inflammation, one-way ANOVA with Bonferroni corrections was performed for multiple group comparisons.

## Results

### PI3K/AKT inhibition enhances hyperoxia-induced ALI *in vivo*

**Fig 1:**
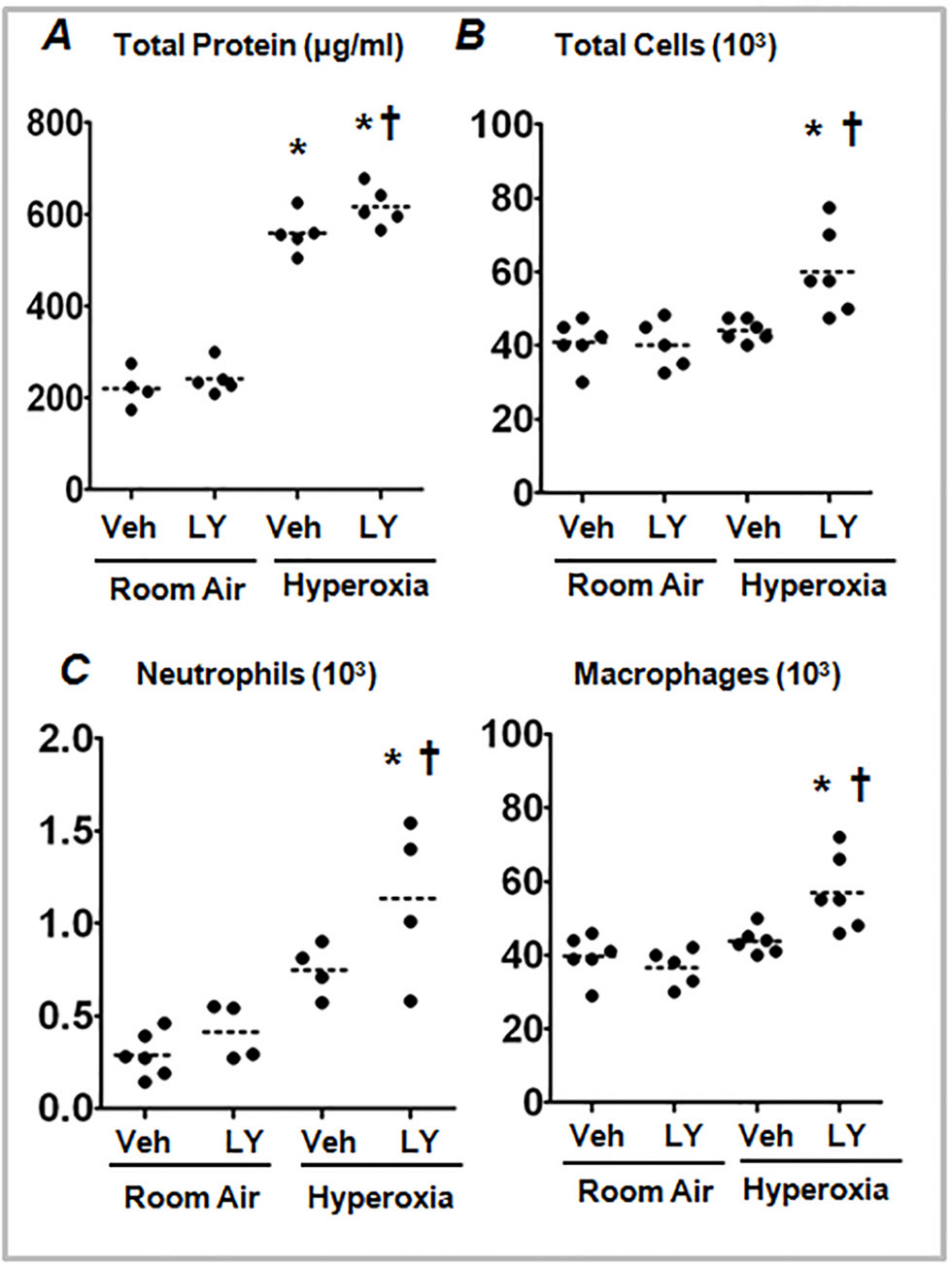
PI3K/AKT inhibition exacerbates hyperoxic lung injury in *Nrf2* ^*+/+*^ mice. Mice were treated *i*.*p*. with LY294002 (LY) or DMSO (vehicle) 1 h prior to exposure and at 24 h intervals during hyperoxia exposure. Mice were exposed to 95% oxygen for 48 h at which time lungs were harvested. The BAL fluid was collected to measure both total cell counts and protein concentration, as markers of lung inflammation and lung injury (permeability), respectively. (A) Total protein in the BAL fluid of room air or hyperoxia-exposed mice treated with vehicle or LY. (B) Total cells in the BAL fluid of room air- or hyperoxia-exposed mice treated with vehicle or LY. (C) Total neutrophils and macrophages in the BAL fluid of vehicle- or LY-treated mice were exposed to room air or hyperoxia. BAL fluids (100 μl) were cytospun onto microslides and stained with Diff Quick stain kit. Differential cell counts were enumerated by counting a total number of 300 cells. The horizontal and vertical lines were plotted as median ± interquartile range for each group (n = 4–5). One-way ANOVA with Bonferroni corrections was performed for multiple group comparisons. *P < 0.05, hyperoxia vs room air; ^***†***^ *P* < 0.05, vehicle vs LY.

To define the role of PI3K/AKT signaling in the regulation of hyperoxia-induced ALI *in vivo*, the wild-type (*Nrf2* ^+/+^) mice were treated with vehicle or PI3K/AKT inhibitor, LY294002, during hyperoxia exposure. Lung injury was evaluated by measuring protein concentration in the BAL fluid, as an indicator of alveolar permeability, whereas lung inflammation was assessed by enumerating the number of inflammatory cells present in the BAL fluid. As shown in Fig 1A, we found a significant increase in the accumulation of total protein in the BAL fluid of mice treated with LY294002 and exposed to hyperoxia, as compared to vehicle-treated counterparts. Likewise, the number of total cells in the BAL fluid was significantly greater in mice treated with PI3K/AKT inhibitor compared to vehicle-treated mice (Fig 1B). The increase in the number of total cells was mainly attributed to the increased accumulation of neutrophils and macrophages (Fig 1C). These results suggest that PI3K/AKT signaling is required for mitigating hyperoxia-induced lung injury and inflammation.

### Inhibition of the PI3K/AKT pathway suppresses Nrf2 target gene expression in the lung

**Fig 2:**
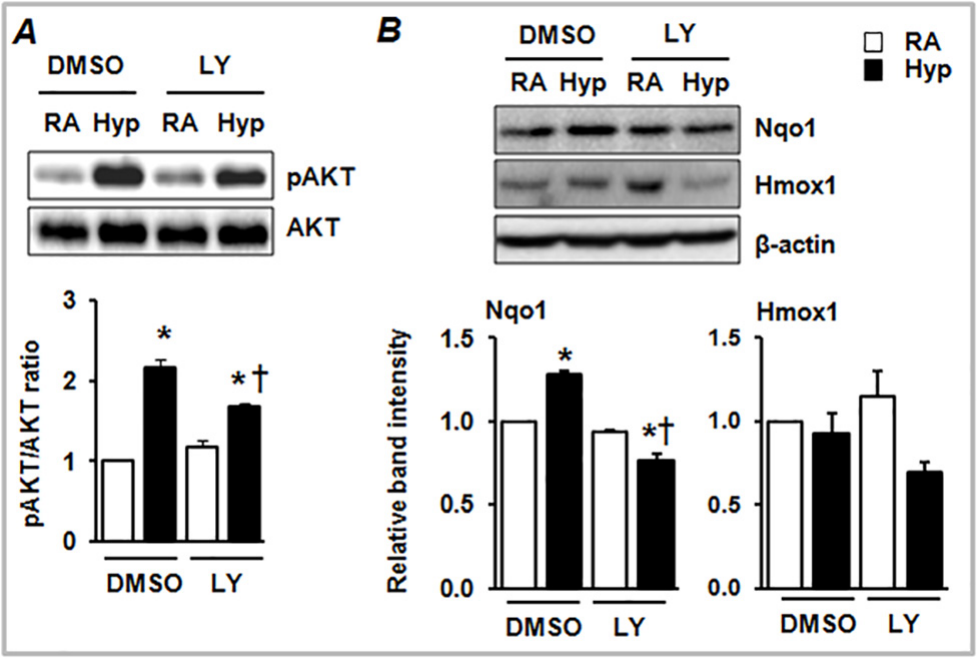
The effects of PI3K/AKT inhibition on Nrf2 target gene expression in mice. Total proteins were extracted from the lungs of vehicle- or LY-treated *Nrf2* ^***+/+***^ and *Nrf2* ^***–/–***^mice exposed to room air or hyperoxia. Equal amounts of proteins were separated and transferred onto PVDF membranes, and probed with anti-pAKT, anti-AKT, anti-Hmox1 or anti-Nqo1 antibodies. β-actin was used as a loading control. The effect of PI3K inhibition on AKT phosphorylation (A) and Nqo1 and Hmox1 expression (B) in the lungs of room air- or hyperoxia-exposed mice. Band intensities were calculated using Image J software and the graphs represent the ratios of pAKT/AKT (n = 4), Hmox1/β-actin (n = 6) and Nqo1/β-actin (n = 6). *P < 0.05, hyperoxia vs room air; ^***†***^ *P* < 0.05, vehicle vs LY.

**Fig 3:**
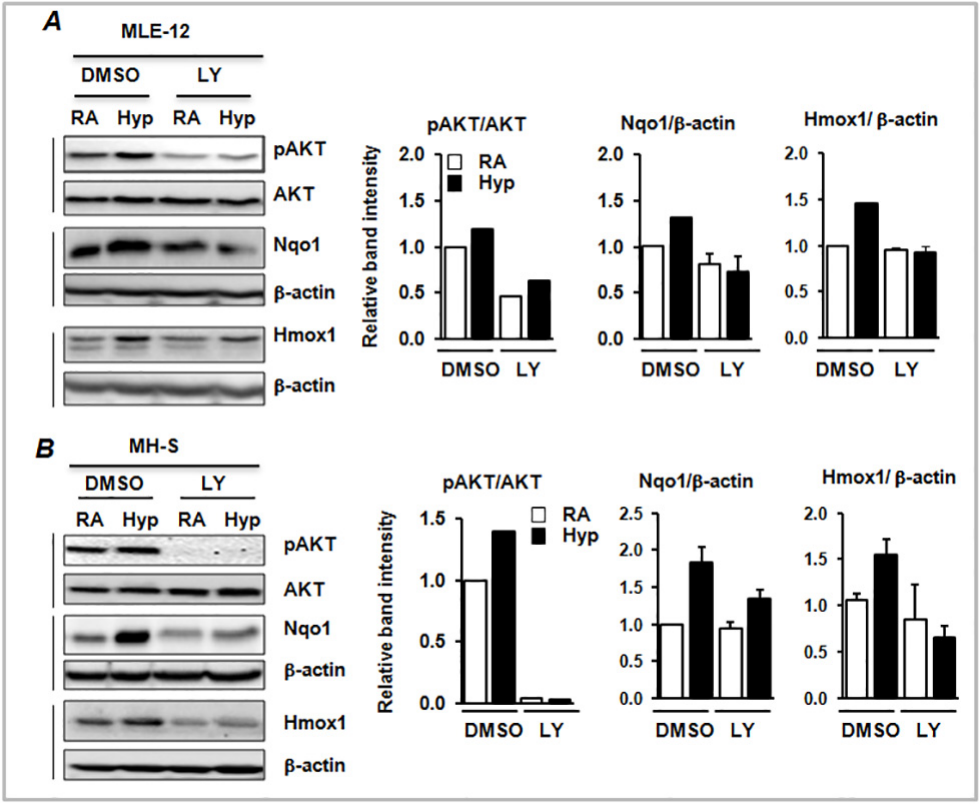
The effects LY29004 on hyperoxia-induced Nqo1 and Hmox1 expression in lung epithelial cells and alveolar macrophages. Total proteins were extracted from vehicle or LY-treated MLE-12 and MH-S cells exposed to room air or hyperoxia for 12 h. Equal amounts of proteins were separated and transferred onto PVDF membranes, and probed with antibodies as indicated.β-actin was used as a loading control. Levels of AKT phosphorylation, and Hmox1 and Nqo1 expression in MLE-12 (A) and MH-S (B) cells treated with vehicle (DMSO) or LY and subsequently exposed to room air or hyperoxia. Band intensities were calculated using Image J software and ratios of pAKT/AKT, Hmox1/β-actin and Nqo1/β-actin. pAKT/AKT levels shown are from a representative experiment. Relative band intensities of Nqo1 and Hmox1 are from two independent samples (n = 2).

To determine the role of PI3K/AKT signaling in regulating Nrf2-regulated anti-oxidant gene expression during hyperoxia, mice were treated with LY294002 and exposed to hyperoxia for 24 h. First, we determined the AKT activation levels in the lung lysates of these mice by immunoblotting with phospho-(Thr^308^) and total AKT1/2 antibodies. As expected, hyperoxia caused the stimulation of AKT1/2 phosphorylation (Fig 2A), but LY294002 significantly inhibited it. To determine whether AKT1/2 inhibition results in decreased Nrf2 antioxidant response, we analyzed the expression levels of Nrf2 targets, heme oxygenase 1 (Hmox1) and NAD(P)H quinone oxidoreductase (Nqo1), by immunoblotting. As shown in Fig 3B, PI3K/AKT inhibition decreased hyperoxia-induced Nqo1 expression. We did not find an increased expression of Hmox1 in the lungs of hyperoxia-exposed mice and PI3K/AKT inhibition did not alter its expression levels.

### PI3K/AKT inhibition dampens Nrf2 target gene expression in lung alveolar epithelial cells and macrophages

To validate the involvement of the PI3K/AKT pathway in the regulation of Nrf2 target (Hmox1 and Nqo1) gene expression during lung injury and inflammation, both mouse lung alveolar epithelial cells (MLE-12) and alveolar macrophages (MH-S) were treated with LY294002 for 30 min and then exposed to room air or hyperoxia for 12 h. AKT1/2 activation and Hmox1 and Nqo1 expression, were analyzed by immunoblotting (Fig 3). Hyperoxia induced AKT1/2 activation in both cell types, but pretreatment of cells with LY294002 inhibited it in both MLE-12 (Fig 3A) and MH-S (Fig 3B) cells. Moreover, LY294002 attenuated hyperoxia-induced Hmox1 and Nqo1 expression, suggesting that PI3K/AKT signaling regulates hyperoxia-induced Nrf2-mediated anti-oxidant gene expression.

### AKT1 knockdown dampens hyperoxia-induced Nrf2 target gene expression in alveolar epithelial cells

**Fig 4:**
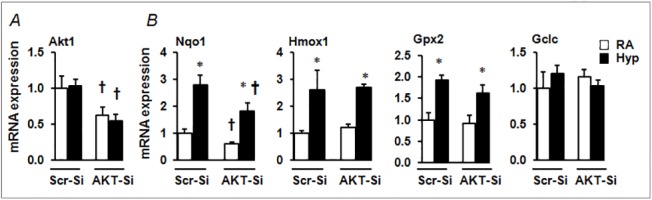
Hyperoxia-induced Nrf2-target gene expression in AKT1-depleted cells. (A) qRT-PCR analysis of hyperoxia-induced Nrf2-target gene expression in cells transfected with either scrambled siRNA (Scr-Si) or AKT1 siRNA. (A) Expression of AKT1 was calculated relative to Scr-Si-transfected room air-exposed samples. (B) Nrf2-target gene expression was calculated relative to Scr-Si-transfected room air-exposed samples. Values from the Scr-si transfected cells exposed to room air are considered as one unit. *p* ≤ 0.05, room air (RA) vs. hyperoxia (hyp); ^***†***^ *p* ≤ 0.05, Scr siRNA vs AKT1-siRNA. Data are expressed as mean ± SEM (n = 3–4).

To determine whether LY294002-exerted effects on Nqo1 expression are mediated through the inhibition of AKT1/2 activation, we performed siRNA-mediated knockdown of AKT1 in MLE-12 cells and measured the expression levels of Nrf2 target genes induced by hyperoxia (Fig 4). Transfection with AKT1 siRNA for 48 h resulted in an approximately 45% decrease in AKT1 mRNA expression in MLE-12 cells (Fig 4A). Knockdown of AKT1 resulted in decreased expression of the Nrf2 target gene, Nqo1, by 40%, which corresponds with the percentage of AKT1 knockdown under basal conditions. Hyperoxia caused a 3-fold increase in Nqo1 expression (Fig 4B). However, hyperoxia-induced Nqo1 expression was significantly lower than control (scrambled)-siRNA transfected counterparts. Hyperoxia-induced Hmox1 expression, but AKT1 knockdown had no significant effect on the expression levels of Hmox1 (Fig 4B). Hyperoxia-induced GPX2 expression was modestly reduced in AKT1 siRNA transfected cells. These results indicate that AKT1 modulates hyperoxia-induced Nrf2 target gene expression in a promoter context-dependent manner.

### PI3K/AKT inhibition causes lung injury and neutrophilic inflammation in *Nrf2* ^–/–^mice

**Fig 5:**
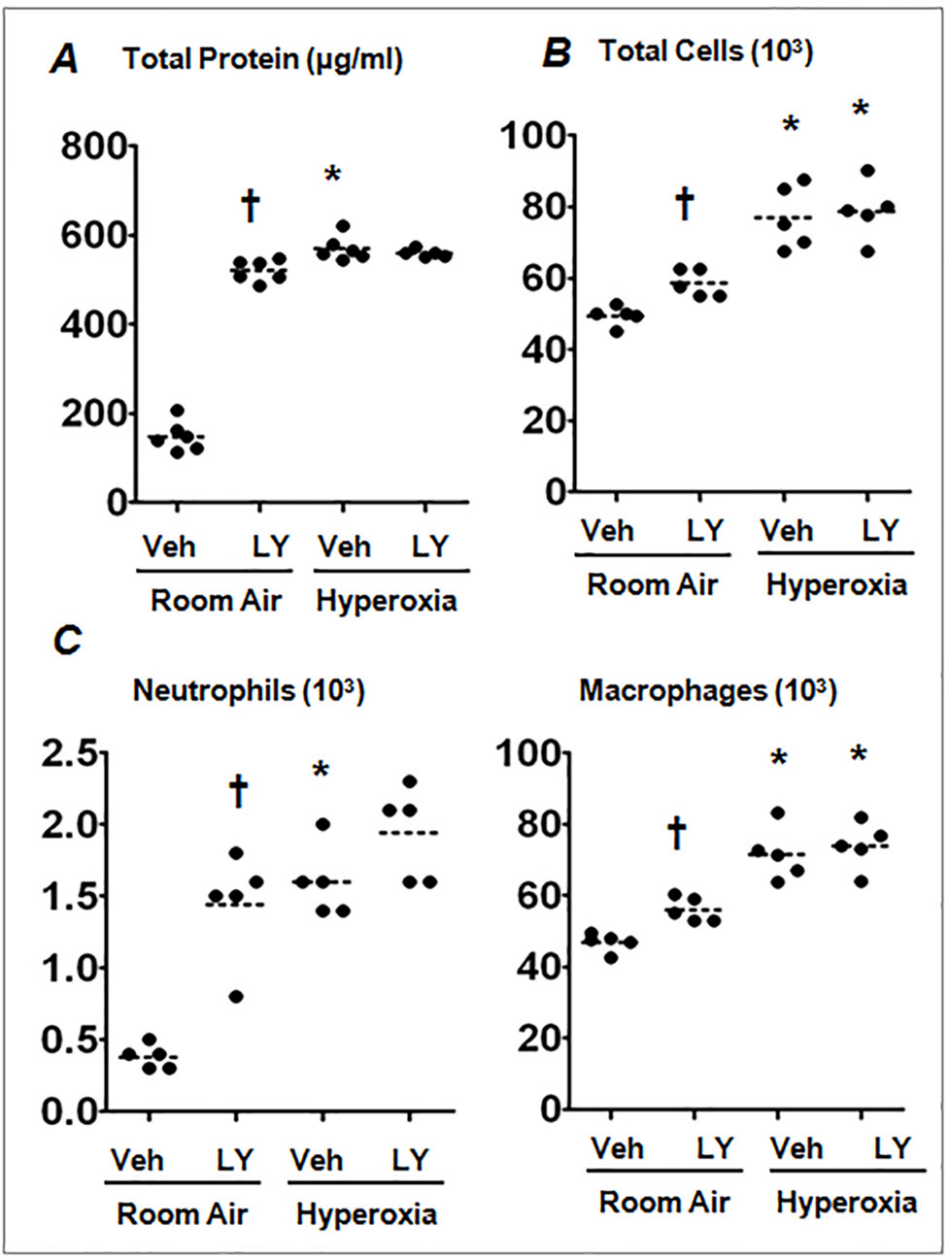
The effects of PI3K/AKT inhibition on hyperoxic lung injury and inflammation in *Nrf2* ^*–/–*^mice. *Nrf2* ^***–/–***^mice were treated with either vehicle or LY and subsequently exposed to hyperoxia or room air as described in Fig 1. (A) Total protein in the BAL fluid of room air or hyperoxia exposed *Nrf2* ^***–/–***^mice treated with vehicle or LY. (B) Total cells in the BAL fluid of room air or hyperoxia exposed *Nrf2* ^***–/–***^mice treated with vehicle or LY. (C) Total neutrophils and macrophages in vehicle or LY-treated mice exposed to room air or hyperoxia. The horizontal and vertical lines were plotted as median ± interquartile range for each group (n = 4–5). One-way ANOVA with Bonferroni corrections was performed for multiple group comparisons. *P = 0.05, hyperoxia vs room air; ^***†***^ *P* = 0.05, vehicle vs LY treated group.

To determine whether PI3K/AKT signaling confers protection against ALI in an Nrf2-dependent manner, *Nrf2* ^–/–^mice were treated with LY294002 and exposed to room air or hyperoxia; lung injury and inflammation were assessed. Unlike in *Nrf2* ^+/+^ mice, PI3K/AKT inhibition had no additive or exaggerated effect on lung alveolar permeability (Fig 5A) and inflammation (Fig 5B & 5C) in *Nrf2* ^–/–^mice exposed to hyperoxia. However, it caused markedly increased total protein accumulation and inflammation, comprising of neutrophils and macrophages, in *Nrf2* ^–/–^mice exposed to room air, as compared to vehicle (DMSO)-treated mice. These results suggest that Nrf2 is required for the PI3K/AKT pathway to impart its protective effects during hyperoxia, but this signaling pathway maintains lung alveolar permeability and inflammation in the absence of Nrf2 under basal condition.

### PI3K/AKT signaling promotes lung inflammation during post-injury

**Fig 6:**
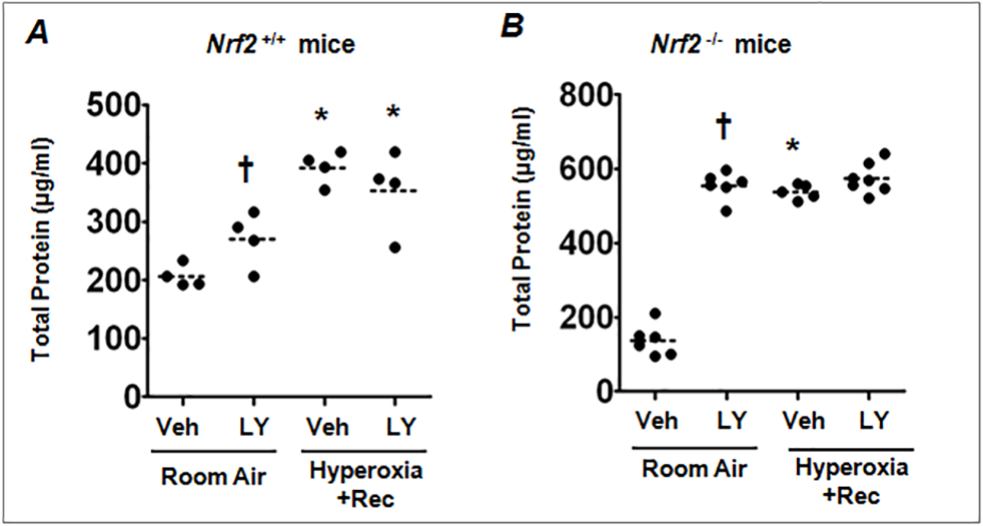
The effects of PI3K/AKT inhibition on hyperoxic lung injury in *Nrf2* ^*+/+*^ and *Nrf2* ^*–/–*^mice post-injury. *Nrf2* ^***+/+***^ and *Nrf2* ^***–/–***^mice were treated with vehicle or LY during hyperoxia as well as post-injury (recovery) from hyperoxia for 72 h at every 24 h intervals. Lung injury and inflammation was assessed as described in Fig 1. Lung alveolar permeability of *Nrf2* ^***+/+***^ (A) and *Nrf2* ^***–/–***^(B) mice post hyperoxic injury. The horizontal and vertical lines were plotted as median ± interquartile range for each group (n = 4–5). One-way ANOVA with Bonferroni corrections was performed for multiple group comparisons. *P = 0.05, hyperoxia vs room air; ^***†***^ *P* = 0.05, vehicle vs LY treated group.

We next determined whether the PI3K/AKT pathway is required to dampen lung injury and inflammation during the recovery from hyperoxic injury, and whether this occurs through an Nrf2-dependent or-independent manner. To evaluate this aspect, *Nrf2* ^+/+^ and *Nrf2* ^–/–^mice were given LY294002 during both 48 h hyperoxia and recovery from injury. Mice were then sacrificed at 72 h post-injury. As shown in Fig 6, BAL protein analysis revealed that persistent PI3K/AKT inhibition either protected or exacerbated lung injury in both *Nrf2* ^+/+^ (panel A) and *Nrf2* ^–/–^(panel B) mice during recovery from hyperoxic lung injury. In fact, LY294002 caused a significant increase in alveolar permeability in both genotypes exposed to room air when compared to vehicle-treated counterparts.

**Fig 7:**
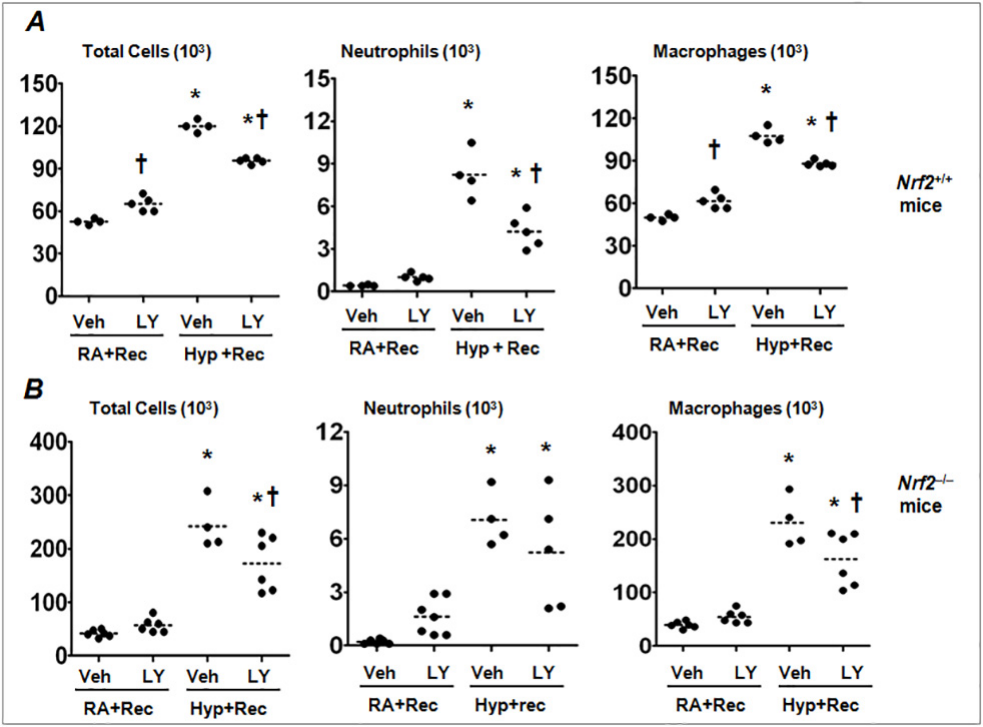
PI3K/AKT inhibition attenuates lung inflammation in *Nrf2* ^*+/+*^ and *Nrf2* ^*–/–*^mice post-injury. Cytospin slides of BAL cells were prepared as described in Fig 1. Total cells, neutrophils and macrophages from room air or hyperoxia exposed *Nrf2* ^***+/+***^ mice (A) and *Nrf2* ^***–/–***^mice (B) treated with vehicle or LY. *P = 0.05, hyperoxia vs room air; ^***†***^ *P* = 0.05, vehicle vs LY treated group.

To determine whether PI3K/AKT signaling affected lung inflammation during post-injury, we enumerated the composition of inflammatory cells in the BAL fluids of these mice. As shown in Fig 7, PI3K/AKT inhibition significantly blocked hyperoxia-induced total inflammatory cell infiltration (Fig 7A, left panel) comprising of neutrophils (middle panel) and macrophages (right panel). However, in room air-exposed uninjured *Nrf2* ^+/+^ mice, PI3K/AKT inhibition resulted in increased number of total cells, mainly attributed to macrophages (Fig 7A, right panel) as compared to room air-exposed vehicle-treated mice. In *Nrf2* ^–/–^mice, unlike 48 h inhibition (see Fig 5), prolonged LY294002 treatment did not alter total inflammatory cell number or composition under basal condition (Fig 7B). However, PI3K/AKT inhibition significantly attenuated macrophagic inflammation and modestly attenuated neutrophilic inflammation in *Nrf2* ^–/–^mice after hyperoxic lung injury (Fig 7B). These results indicate that the PI3K/AKT signaling pathway promotes inflammation in pre-injured lungs during recovery, while suppressing the inflammation in uninjured lungs.

## Discussion

Nrf2-regulated redox balance has been shown to be perturbed in several lung diseases, including ALI [20, 21]. Previously, we have identified Nrf2 as a candidate susceptibility gene for hyperoxia-induced ALI [6]. We have also shown that *Nrf2*-deficient mice are more susceptible than wild-type mice to lung inflammatory and hyper-permeability responses induced by hyperoxia [7, 8], and that they show diminished constitutive and inducible antioxidant gene expression [6, 7]. The PI3K/AKT signaling pathway also confers protection against oxidant-induced ALI [13]. Although we have demonstrated that PI3K/AKT signaling is required for Nrf2 activation by hyperoxia in lung epithelial cells [11], it is unclear whether PI3K/AKT-Nrf2 crosstalk plays a protective role during lung injury and repair. In this context, the present study demonstrated that PI3K/AKT-Nrf2 signaling is required to dampen hyperoxia-induced lung injury and inflammation *in vivo*.

The PI3K/AKT pathway regulates a number of biological processes including cell growth, differentiation, apoptosis and neoplastic transformation [10, 22–24]. We found that inhibition of the PI3K/AKT pathway exacerbates lung alveolar permeability and leukocyte inflammation (Fig 1). PI3K/AKT inhibition resulted in diminished levels of hyperoxia-induced Nqo1 expression, an Nrf2-target gene, *in vivo* (Fig 2) and in alveolar type II-epithelial cells and macrophages *in vitro* (Figs 3 and 4). Given that the Nrf2-deficiency enhances cell death, and its overexpression confers cellular protection against pro-apoptotic stimuli [25, 26] including hyperoxia [27], we propose that the PI3K/AKT pathway modulates cell death and subsequent inflammatory pathways via the Nrf2-ARE mediated antioxidant transcriptional response. Previously, we have shown that P13K/AKT signaling is required for nuclear accumulation of Nrf2 in lung epithelial cells exposed to hyperoxia. Glycogen synthase kinase 3β (GSK3β) is a major downstream target of PI3K/AKT signaling pathway and phosphorylates various substrates [28, 29]. Recently, it was reported that GSK3β -mediated phosphorylation of Nrf2 causes the nuclear exclusion of this transcription factor and the down-regulation of ARE transcriptional activity [30]. As AKT1/2 phosphorylates GSK3β and inactivates its enzyme activity, it is unclear whether AKT1/2 increases Nrf2 nuclear accumulation by phosphorylating Nrf2 directly and/or by inactivating GSK3β activity in response to hyperoxic stress; this warrants further studies.

Another important finding of our study is that inhibition of PI3K/AKT pathway causes lung injury and inflammation in the setting of Nrf2-deficiency under basal condition (Fig 5). Lung alveolar permeability and inflammatory cell infiltration in *Nrf2* ^–/–^mice with PI3K/AKT inhibition were induced to a similar extent to that of vehicle-treated *Nrf2* ^–/–^mice exposed to hyperoxia (Fig 5). Previously, we have shown that Nrf2 deficiency results in increased levels of oxidative stress and cell death [31], and activation of AKT1/2 signaling in response to growth factors such as insulin and PDGF is impaired in Nrf2-deficient primary alveolar type II epithelial cells [32]. Thus, it is likely that the PI3K/AKT pathway inhibition in absence of Nrf2 may cause alveolar epithelial cell dysfunction, leading to increased alveolar permeability and inflammation *in vivo*. However, PI3K/AKT inhibition did not cause either synergistic or additive effects on hyperoxia-induced lung injury in *Nrf2*-deficient mice (Fig 5), suggesting that PI3K/AKT signaling protects hyperoxia-induced ALI through Nrf2. Several studies have shown that oxidative imbalance correlates with the perturbed activity or stability of Nrf2 protein [33–36]. Consequently, hyperoxia-induced lung injury and inflammation may depend on the prevailing stress levels and the subsequent signaling mechanisms activated by them. Further studies are warranted to show how the PI3K/AKT pathway imparts its protective functions in the settings of impaired Nrf2 signaling and how it maintains lung homeostasis under basal conditions.

Paradoxically, PI3K/AKT pathway also promotes lung inflammation, independent of Nrf2, after injury. Our studies also revealed that PI3K/AKT inhibition post-hyperoxic injury dampens inflammatory cell infiltration into the lungs, but it did not improve lung injury, i.e., the accumulation of total protein in the alveolar space. These results suggest a regulatory role for PI3K/AKT pathway in modulating lung inflammation during the resolution of ALI. Recruitment of inflammatory cells and subsequent generation of oxidants, proteases and inflammatory cytokines are the key elements in the pathogenesis of ALI [37, 38]. The PI3K/AKT pathway promotes Th2-cytokine mediated eosinophilic infiltration, mucous production and airway hyper-responsiveness in a mouse model of experimental asthma [19]. Likewise, inhibition of PI3K-γ dampens LPS-induced lung leukocyte infiltration and inflammatory cytokine expression via NF-κB [16]. In our experimental settings, PI3K/AKT inhibition in mice recovering from injury decreased leukocyte (neutrophilic and macrophage) accumulation in wild type (Fig 6). It also decreased macrophage and neutrophil accumulation in *Nrf2* ^–/–^mice (Fig 7), suggesting that the PI3K/AKT pathway promotes lung inflammation independent of Nrf2 status post-injury. Clearance of the debris, dead cells and other soluble materials by macrophages in the lung are critical to modulate the local inflammatory responses and proper resolution of lung tissue repair [39, 40]. Thus, it is possible that the PI3K/AKT pathway may regulate the steady state levels of phagocytes in the lung until the complete cessation of lung inflammation in order to maintain tissue homeostasis, and this warrants further studies.

It is noteworthy that not all Nrf2 target gene expression induced by hyperoxia is modulated by either PI3K/AKT-mediated chemical inhibition or si-RNA-mediated AKT1 depletion in our experimental setting (Figs 3 and 4), suggesting that PI3K/AKT signaling selectively regulates Nrf2-dependent AOE expression in response to oxidant stress. In support of this contention, it has been reported that Nrf2 binds to the ARE in a promoter-specific manner in mammary epithelial cells in response to arsenite-induced stress [41]. It is important to note that other transcription factors such as AP-1 and p53 proteins regulate antioxidant gene expression, and thus it is possible that these proteins may be involved in regulating antioxidant (i.e., Hmox1) gene expression in the deficiency of PI3K/AKT-mediated Nrf2 signaling under our experimental conditions.

In summary, our data show that the PI3K/AKT signaling pathway confers protection against hyperoxia-induced ALI by regulating Nrf2-mediated ARE transcriptional activity in a promoter-context dependent manner, and that it also maintains lung homeostasis in settings of Nrf2 deficiency. In addition, we identified that the PI3K/AKT pathway promotes macrophage recruitment into the lung during the resolution phase of injury independent of Nrf2 status.
